# Therapeutic management of complicated talar extrusion: literature review and case report

**DOI:** 10.1007/s10195-011-0129-z

**Published:** 2011-02-25

**Authors:** Luca Vaienti, Francesco Maggi, Riccardo Gazzola, Edoardo Lanzani

**Affiliations:** 1 Dipartimento di Scienze Medico Chirurgiche, Università degli studi di Milano; IRCCS Policlinico San Donato, Piazza Malan, 20097 San Donato Milanese, Milano, Italy; 2 Istituto Ortopedico “Gaetano Pini”, P.zza Ferrari 1, 20122 Milan, Italy; 3 Plastic Surgery Department, Università degli Studi di Milano, Policlinico San Donato, Piazza Malan, 20097 San Donato Milanese, Milan, Italy

**Keywords:** Total talar dislocation, Talar extrusion, Missing talus, Distally-based sural fasciocutaneous flap

## Abstract

Total extrusion of the talus with interruption of all ligaments (missing talus) is a rare injury. We describe the case of a 27-year-old man who reported total extrusion of the talus after a motorbike accident with interruption of all talar ligaments. In the first repair effort, the articular void left by the talus was filled with antibiotic cement and the wound was closed primarily. Nevertheless, the skin overlying the talar joint displayed necrosis. In order to cover the cutaneous defect, improve local vascularization, and allow reimplantation of the talus, a sural fasciocutaneous island flap was harvested. Subsequently, the original talus was placed and arthrodesis of the subtalar joint was performed. The patient was able to walk bearing full weight without support equipment after 6 months. Several therapeutic options have been suggested in such cases, including replacing the talus, tibiocalcaneal arthrodesis, and pseudoarthrodesis. The rarity and peculiarity of such cases make the establishment of generalized guidelines an arduous task, leaving the choice of treatment to the surgeon, in conformity with each case’s peculiarity. In this case use of the flap may have promoted the vascularization of the reimplanted talus, thus avoiding avascular necrosis and allowing successful reimplantation of the original talus.

## Introduction

Total dislocation and extrusion of the talus is a rare injury produced by an excessive tibiotalar dorsiflexion or plantarflexion in combination with subtalar supination or pronation in high-energy traumas [1]. Talar dislocation is a well-known lesion, but the majority of these cases consists of open fractures and dislocations in which the talus preserves at least one capsular bond. In these injuries, the talus loses its anatomical relationships with tibia, calcaneus, and navicular bone, thus impeding all weight-bearing movements [2]. Recently, large case series suggest talar reimplantation after total extrusion with variable preservation of ligaments, showing acceptable infection rates and rare talar body collapses [3].

When the talus loses all soft-tissue attachments, it is called missing talus. Although several cases of total talar extrusion have been reported in literature, we found only six cases of complete talar extrusion with loss of all ligaments [4–8]. Considering the absence of guidelines, managing this condition is supported by surgical experience and few cases of class V evidence in the literature. On the other hand, the complications of these injuries could be compared with those of complete talar extrusion without loss of talar ligaments and to talar neck fractures. Complications could be divided in two categories: short-term (infection) and long-term complications. Although no data about infection rates in missing talus injuries are available, the likelihood of infection is strictly correlated with open fracture and extrusion. Marsh reports infection in seven patients (38%) in the treatment of 18 open fracture dislocations [9], whereas considerably lower rates are reported by Smith and colleagues [3], who report one case of infection out of 27 cases of extruded talus (4%). Long-term complications could be observed somewhere about 12 months after trauma and consist of bone collapse, stiffness, arthritis, and bone necrosis. In consideration of the loss of all ligaments and vascular supply, bone necrosis is the most dreaded complication in missing talus accidents.

In this case report, we propose an original approach characterized by the use of antibiotic cement, followed by harvesting of a sural fasciocutaneous flap and reimplantation of the original talus. The sural fasciocutaneous flap is able to bring excellent trophism to the acceptor site and may be successfully employed to treat defects of the proximal third of the foot and the lower leg [10–13].

## Case report

**Fig. 1:**
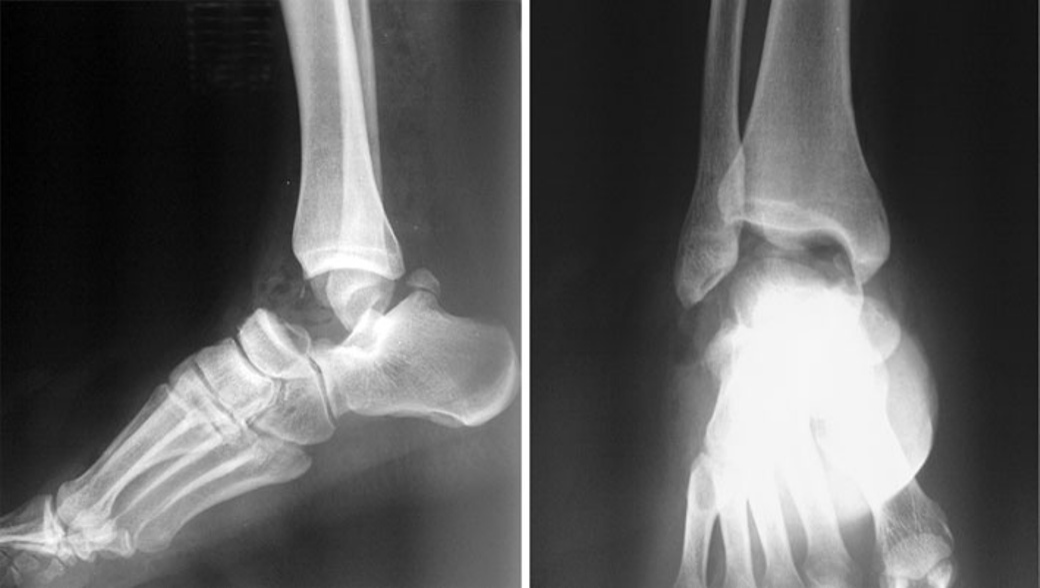
X-ray shows the absence of talus without any fracture of the surrounding bones

A 27-year-old man was brought into the emergency department after a motorbike accident. No wounds were reported other than the total extrusion of the right talus (Fig. 1). The bone pedicle appeared severely damaged, whereas the talar bone was intact and was stored in a bone bank. Irradiation was employed to preserve and sterilize the bone.

**Fig. 2:**
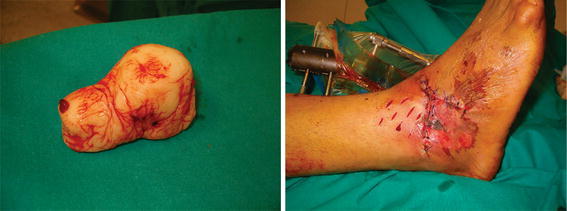
Properly modeled antibiotic cement was employed to fill the talar void (*left*). An external fixator was employed to stabilize the joint; the wound was closed primarily (*right*)

**Fig. 3:**
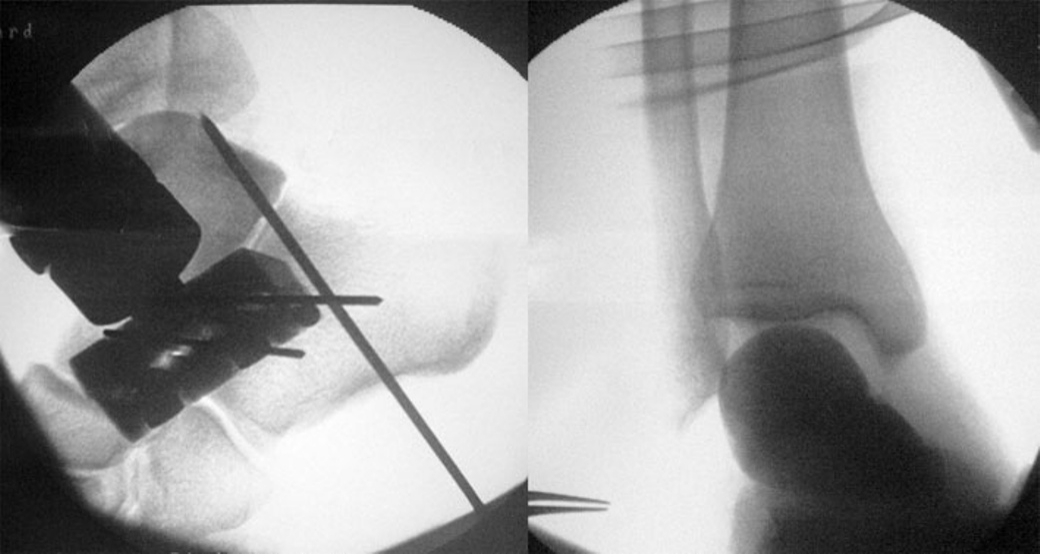
Intraoperative X-rays after application of antibiotic cement

A culture was obtained and empiric antibiotic therapy with teicoplanin and amikacin was started. The lacerated wound was copiously irrigated, cleansed with povidone iodine, and debrided. The talar void was filled with properly modeled gentamicin/clindamycin-loaded cement spacer in order to reduce the risk of articular talar space loss and infection while waiting the cultural results (Figs. 2, 3). An external fixator, with pins inserted in the tibia and calcaneus, was applied to stabilize the joint. Vascularization of the foot appeared intact; tendons and muscles did not display damage; no neurological deficit was reported; and the skin defect was closed primarily.

**Fig. 4:**
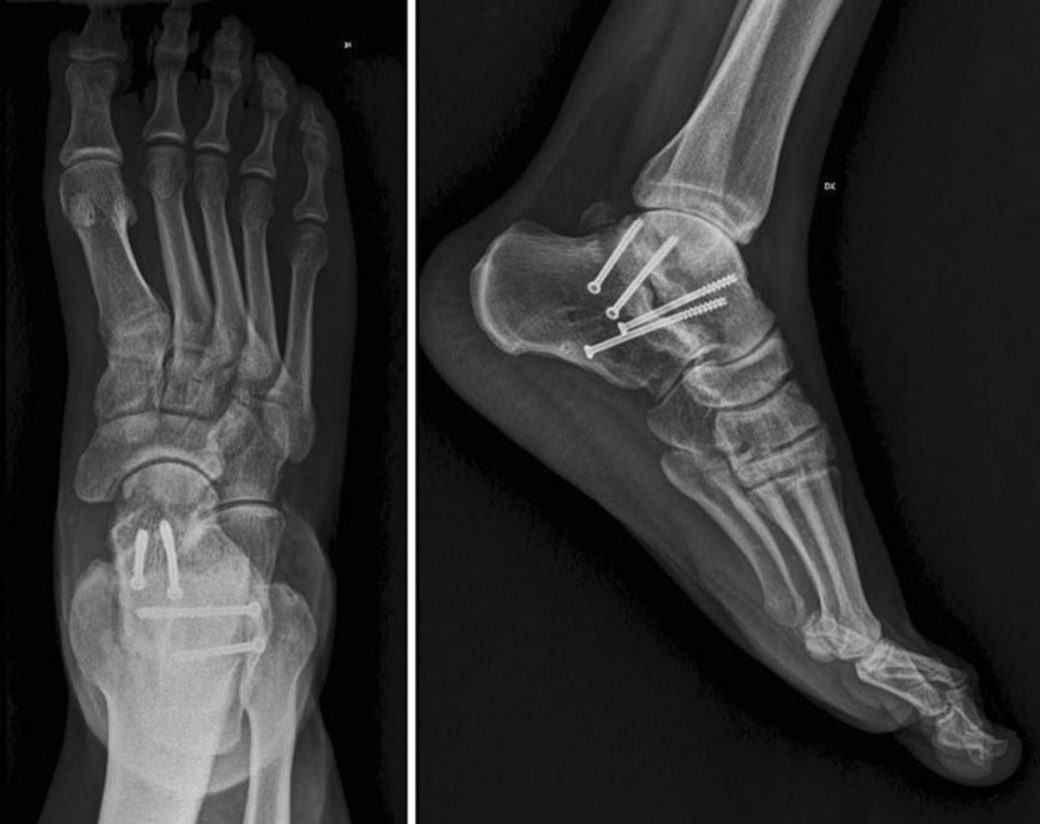
Arthrodesis with two screws in the anterior subtalar joint and in the posterior subtalar joint. Anteroposterior and lateral X-ray projections after 2 years of follow-up. No signs of avascular necrosis are observed

After 2 weeks, the cutaneous margins of the wound and the surrounding skin displayed necrosis. The patient was referred to our department of plastic surgery. The cultures gave negative results. After debridement of the necrotic tissue, the cutaneous defect measured 6 × 5 cm. The antibiotic cement was removed, and two polyvinyl alcohol sponges were placed in the articular void in order to prevent any damage to the articular cartilages. During the same surgical time, a distally based sural fasciocutaneous flap measuring 8 × 6 cm was harvested and applied to the defect. Split-thickness skin grafts were employed to cover the flap pedicle. Twenty-five days later, the external fixator was removed and the original talus was placed, with a dorsal incision 2 cm above the flap margin. Arthrodesis was performed percutaneously using two screws in the anterior subtalar joint and two in the posterior subtalar joint (Fig. 4).

**Fig. 5:**
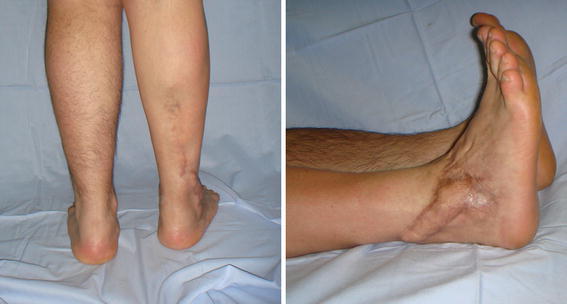
Final result after 4 years. Movements are not impaired; ambulation is regular

Mobilization of the ankle without weight bearing was allowed after removal of the external fixator. Literature is rather unforthcoming in suggesting guidelines due to the rarity of missing talus lesions. We opted to precociously allow careful movements without weight bearing in order to avoid atrophy and to restore talus and surrounding tissues. Weight bearing was then gradually introduced at 3–4 months after the final surgery, and after 6 months, full weight bearing was achieved. At the 4-year follow-up, the joint showed no signs of avascular necrosis, neither plantarflexion nor dorsiflexion showed impairment, and ambulation was regular (Fig. 5).

## Discussion

Talar extrusion is a rare injury, and only few cases have been reported in literature. Management of total talar extrusion could be divided in two types. In the first type, the reconstructive surgeon has to avoid infection and prevent permanent damage to the articulation. Lee [2] suggests immediate reimplantation of the talus in the event that vascular supply is not permanently damaged. Often, this strategy is recommended owing to the possibility of infection, which can affect 38–85.7% of cases [14]. In consideration of infective and necrotic risks, which are higher in cases of immediate talus reimplantation, we delayed reimplantation. The talus-shaped antibiotic cement was employed while waiting for cultural results and to reduce the risk of articular talar space loss.

In a second phase, reconstruction was performed. Several reconstructive options should be considered, including replacing the talus, tibiocalcaneal arthrodesis, and pseudoarthrodesis. Marsch, in his retrospective study of 27 open extrusions of the talus (without interruption of vascular pedicles), observed that the favorable outcomes could be explained by a protocol including serial irrigation, debridement, and rigid fixation [9], whereas more recent studies demonstrate that reimplantation has proven to be a safe treatment [3]. In our case, although the vascular supply was completely interrupted, reimplantation of the talus with arthrodesis of the subtalar joint was performed. This treatment was supported by the use of a sural fasciocutaneous flap which increased the vascular supply to the traumatized area and reduced the otherwise high risk of avascular necrosis of the reimplanted talus. Although the vascular supply was interrupted, no signs of avascular necrosis of the bone were observed in 4 years. Managing such cases remains controversial, and good options are talus replacement, tibiocalcaneal arthrodesis, and pseudoarthrodesis. Notwithstanding, the successful outcome in our case strongly supports our therapeutic choices.
